# Effect of preoperative liraglutide 3.0 mg on incidence of intraoperative adhesions in laparoscopic sleeve gastrectomy

**DOI:** 10.1007/s00464-024-11231-w

**Published:** 2024-09-30

**Authors:** G. Martines, C. Giove, B. Carlucci, A. Dezi, C. Ranieri, M. T. Rotelli, M. De Fazio, G. Tomasicchio

**Affiliations:** 1 Azienda Ospedaliero Universitaria Policlinico, University of Bari, Piazza G Cesare, 11, 70124 Bari, Italy; 2 General Surgery Unit “M. Rubino”, DiMePRe-J, University of Bari Aldo Moro, Bari, Italy; 3 Department of Precision and Regenerative Medicine and Jonic Area (DiMePRe-J), DiMePRe-J, University of Bari Aldo Moro, Bari, Italy; 4 Centro Interdipartimentale di Ricerca sulle Disfunzioni del Pavimento Pelvico, University of Bari Aldo Moro, Bari, Italy

**Keywords:** Obesity, Liraglutide, Sleeve gastrectomy, Adhesions, Weight loss, Post-operative complications

## Abstract

**Introduction:**

Liraglutide has shown promising results in the field of bariatric surgery, preparing patients for surgery. However, chronic therapy is often correlated with gastrointestinal disorders, such as subclinical pancreatitis. The aim of this study was to evaluate the incidence of intraoperative adhesions and post-operative complications in patients undergoing laparoscopic sleeve gastrectomy (LSG) with or without prior therapy with liraglutide.

**Methods:**

Clinical records of patients affected by obesity who underwent LSG between March 2017 and October 2022 were retrospectively reviewed using a prospectively maintained database. Patients were separated into two groups: those managed with preoperative liraglutide for 24 weeks prior to LSG, and those without prior medical therapy. Demographic data, operative time, intraoperative adhesions, and postoperative complications were reported and compared between two groups.

**Results:**

Ninety-three patients underwent LSG without prior medical therapy, while 87 were treated with liraglutide before surgery. There were no significant differences in terms of gender, age, and comorbidities. After treatment with liraglutide, weight (117 vs 109 kg) and BMI (45 vs 42.2 kg/m^2^) were statistically lower than the group with no prior treatment to surgery. Thirty-two (37%) patients of the group treated with liraglutide had intraoperative adhesion vs nine (10%) patients of the control group (*p* < 0.005). There were no differences recorded between the two groups concerning post-operative complications.

**Conclusion:**

Liraglutide has introduced a new way to treat obesity, improving weight loss and comorbidities. Gastrointestinal disorders, such as subclinical pancreatitis, associated with GLP-1 analogue could explain the elevated incidence of intraoperative adhesions during bariatric surgery.

Obesity has become a major public health concern worldwide, with a continuing increasing incidence in western countries. It is associated with numerous comorbidities, including type 2 diabetes, cardiovascular disease, dyslipidemia, respiratory disease and cancers [1]. Bariatric surgery has emerged as a highly effective treatment for obesity, leading to sustained weight loss, improvement in obesity-related comorbidities and quality of life [2]. However, the surgical risk increases progressively according to the body weight and the presence of associated comorbidities [3]. For that reason, lowering the BMI before surgery could greatly help the surgeon to minimize postoperative complications.

Liraglutide, an acylated GLP-1 analogue, produced by recombinant DNA technology has been shown to be able to significantly decrease the body weight and associated comorbidities and therefore could be a useful medical treatment in patients candidate to bariatric surgery [4, 5]. Several studies have evaluated the use of liraglutide as a preoperative therapy to optimize obese patients for surgery, reducing postoperative complications and surgeon discomfort [6]. Nevertheless, chronic therapy with liraglutide 3.0 mg is also correlated with gastrointestinal disorders including pancreatitis, vomiting, nausea, and diarrhea [7, 8]. In particular subclinical pancreatitis can often go undetected because symptoms free and nevertheless they can cause adhesion formation with the posterior gastric wall, making the mobilization of the stomach more complicated and technically demanding.

The aim of this retrospective study was to evaluate the incidence of intraoperative adhesions and their consequences, in patients undergoing laparoscopic sleeve gastrectomy (LSG) with or without preoperative treatment with liraglutide 3.0.

## Materials and methods

A retrospective observational not blinded single-centre study was carried out using a prospectively maintained database of obese patients undergoing LSG in a tertiary referral centre for bariatric treatment between March 2017 and October 2022. Patients over 18 years of age, with a body mass index (BMI) > 40 or > 35 kg/m^2^ with at least one obesity-related comorbidity, such as hypertension, dyslipidemia, or diabetes, who were considered medically fit for surgery with or without preoperative treatment by liraglutide who completed a minimum follow-up period of 12 months, were included in the study. Liraglutide was administered in patients with preoperative BMI > 47 kg/m^2^ with at least one obesity-related comorbidity to optimize patients for surgery, after multidisciplinary evaluation.

Exclusion criteria were the prior use of GLP-1 agonist or family history of medullary thyroid carcinoma, personal or family history of multiple endocrine neoplasia, past history of pancreatitis, acute pancreatitis, alteration of amylase/lipase normal values, liver dysfunction (AST/ALT > 3 times normal value), renal dysfunction (eGFR < 45 mL/min/1.73 m^2^), previous abdominal surgery, acute coronary syndrome in the previous 6 months, intolerance or allergy to liraglutide, discontinuation of liraglutide therapy, pregnancy or breastfeeding, patients with a large hiatal hernia or > 5 cm hernia or ≤ 5 cm with associated severe or intractable gastro-oesophageal reflux symptoms. During the preoperative workup, all patients underwent routine blood test, cardiological, pneumological, psychiatric examination and esophagogastroduodenoscopy in accordance with the European Association for Endoscopic Surgery and Italian Society of Bariatric Surgery recommendations [9]. All the patients provided informed consent for surgery after thorough explanation and counselling of the benefit-risk ratio. Patients fulfilling the inclusion criteria were divided into two non-randomized groups. The first group included obese patients treated with LSG without prior medical therapy, while patients in the second group were treated with liraglutide before LSG. Liraglutide was administered subcutaneously by a multidose pen injector with a starting dose of 0.6 mg/day. The dosage was increased weekly by 0.6 mg up to the maximal dose of 3 mg until 24 weeks. Only patients who reached the maximal dose were included into the study. Patients were trained to self-administer the injection. Demographic data were recorded, including age, gender, weight (kg), BMI (kg/m^2^), and comorbidities. The operative time and the occurrence of intraoperative adhesions between the posterior gastric wall and pancreas were recorded. Intraoperative adhesions were considered positive in case of grade 3 of degree of adhesion (requiring sharp dissection) following the “Modified Leach Grading of Adhesion” [10, 11].

Postoperative complications of LSG were also recorded and scored according to the Clavien-Dindo classification [12]. During post-operative follow-up none of the patients underwent liraglutide therapy.

At 12-months follow-up, all patients underwent outpatient evaluation and were assessed for weight and BMI. Permission for the conduct of this retrospective study was provided by the local hospital Ethics Committee. All investigations complied with the principles of the Declaration of Helsinki.

## Statistical analysis

Continuous parameters were reported as median and interquartile ranges. Categorical variables were recorded as mean and percentages. Comparison of categorical variables was performed by the Chi-squared test and Fisher’s exact test where appropriate. Comparison among groups was made by the Mann–Whitney U test. A *p*-value < 0.05 was considered statistically significant. Statistical analysis was carried out using RStudio (R version 4.0.3 10/ 10/2020 Copyright© 2020, The R Foundation for Statistical Computing).

## Results

**Fig. 1:**
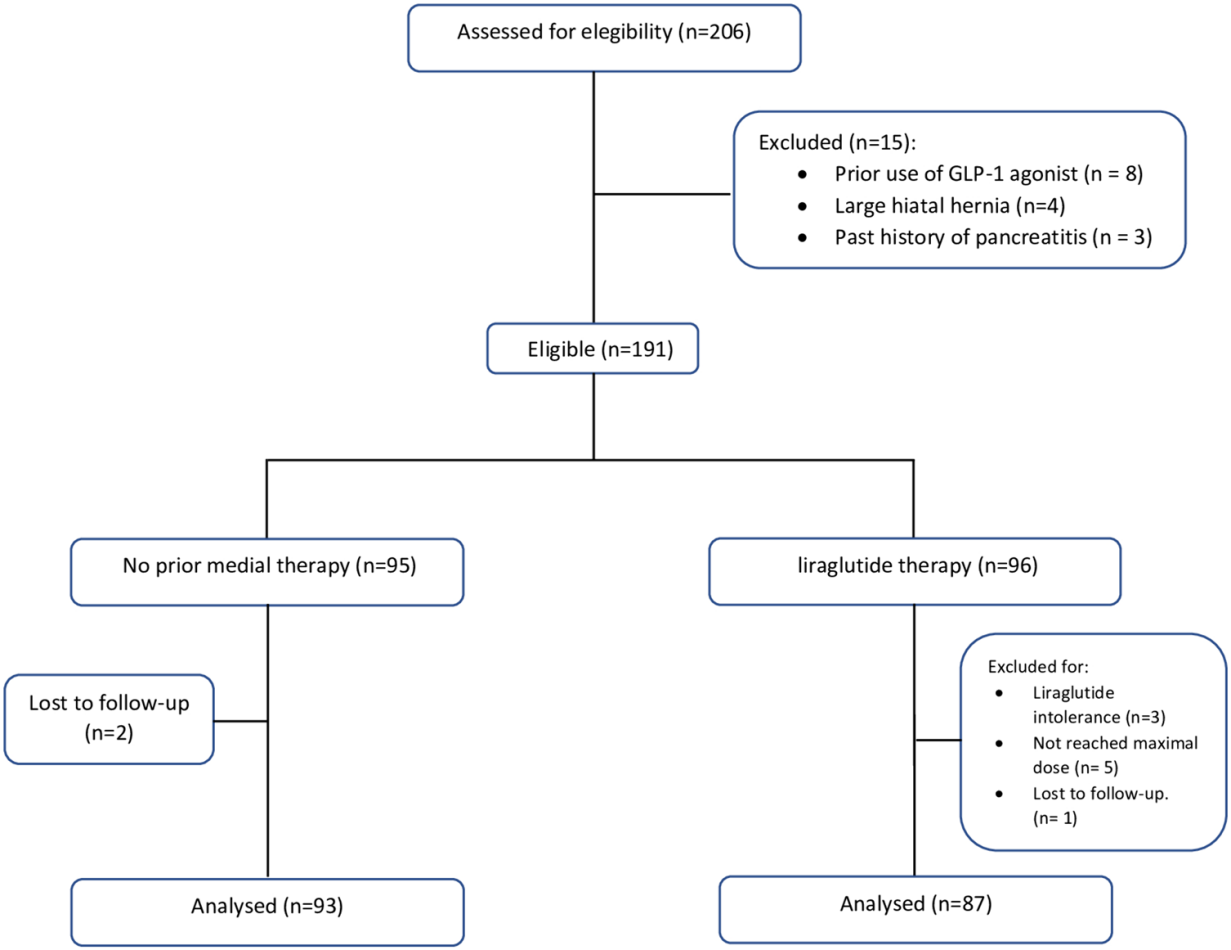
Included patients flowchart (GLP-1: glucagon-like peptide-1, LSG: laparoscopic sleeve gastrectomy)

**Fig. 2:**
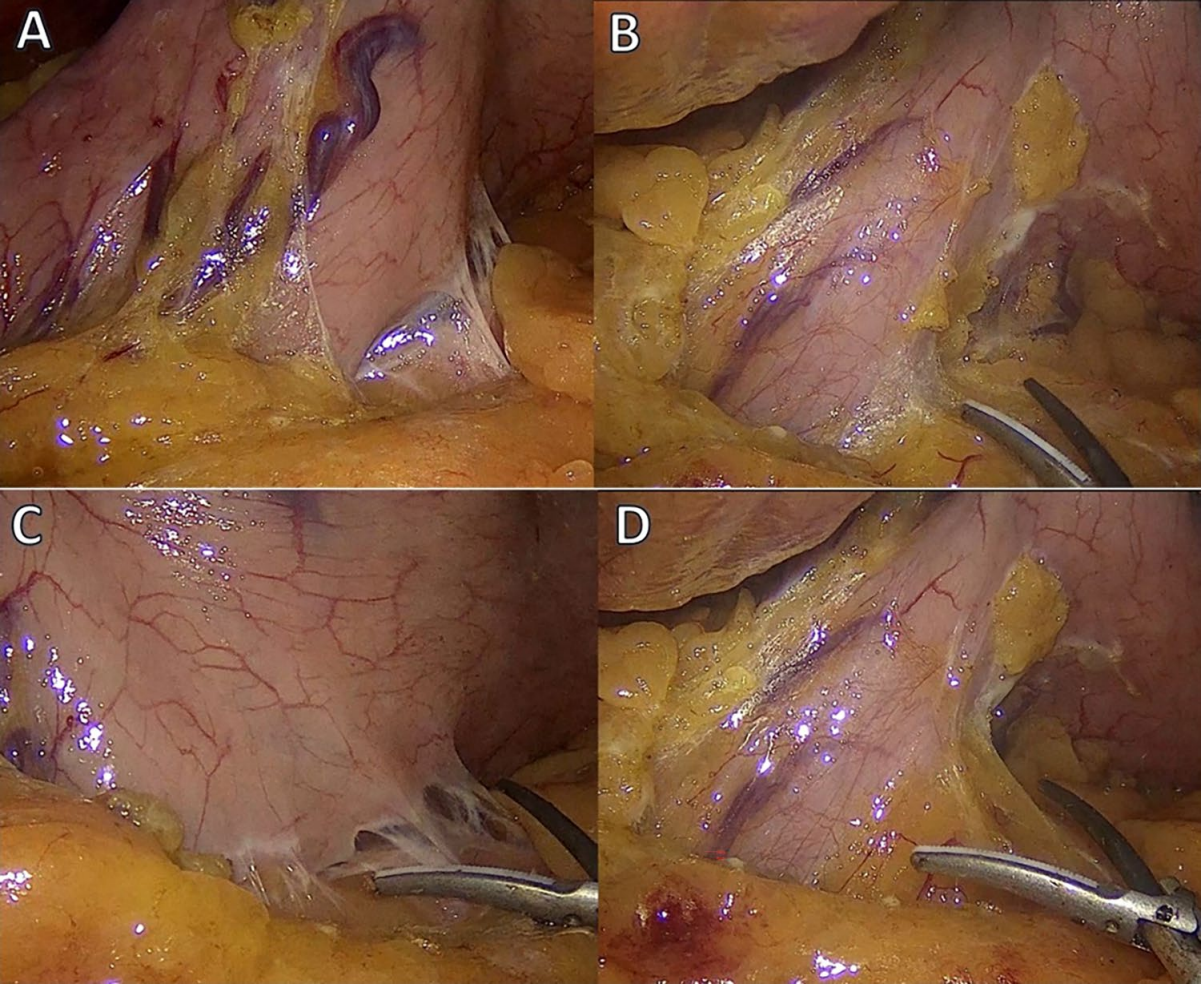
**A**–**D** Intraoperative adhesion between posterior gastric wall and pancreas

A total of 206 obese patients were treated by LSG during the study period. Eight patients were excluded for prior use of GLP-1 agonist, 4 for large hiatal hernia and 3 for history of pancreatitis. In the group treated with liraglutide prior to LSG, nine patients were excluded from the analysis: 3 for liraglutide intolerance, 5 not reaching the maximal dose and only one patient was lost to follow-up. Ninety-three (median age 43 years, IQR 38.5–50, 80% women) of them (51.6%) underwent LSG without prior medical therapy with liraglutide, while eighty-seven (median age 41 years, IQR 35.5–48, 65% women) were treated with liraglutide 3.0 therapy before surgery (Fig. 1). There were no significant differences for gender, age, and comorbidities such ss diabetes mellitus. All patients were affected by grade 3 obesity (BMI ≥ 40 kg/m^2^). At the end of 24 weeks of treatment with liraglutide 3.0, the median preoperative weight and BMI were significantly lower, compared to the group treated only with LSG Table 1. In all patients, sleeve gastrectomy was performed laparoscopically. No conversion to open surgery was required. Thirty-two (37%) patients in the group treated with liraglutide had significant adhesion between the posterior gastric wall and pancreas compared to nine (10%) patients in the control group (*p* < 0.005) Fig. 2. The duration of surgery was statistically longer in the liraglutide group with a median time of 95 min (IQR 88–110) vs 82 min (IQR 78–98) of the control group (*p* < 0.005) (Table 2). There were no differences recorded between the two groups concerning post-operative complications according to Clavien-Dindo grade (Table 3). The three leaks recorded in the group treated with medical therapy before surgery and the one in the control group were all successfully managed by laparoscopic peritoneal lavage and drainage of the subdiaphragmatic space. No postoperative mortality was reported. All patients completed 12 months of follow-up with a median time of 60 months (IQR 55–70), without statistical difference between the two groups. At 12 months follow-up the liraglutide group reported a significantly lower BMI (32.1 kg/m^2^, IQR 29.3–35.8 vs 35.2 kg/m^2^, IQR 30.3–38; *p* < 0.05) compared to the control group.

**Table 1:** Patient pre-operative characteristics

	Liraglutide *n* = *87*	No Liraglutide *n* = *93*	***p-value***
Sex M F	22 (25%) 65 (75%)	19 (20%) 74 (80%)	0.54
Age (years)	41 (35.5 – 48)	43 (38.5 – 50)	0.43
Weight (Kg)	109 (105.5 – 113)	117 (104.5 – 125.5)	< 0.005
BMI (Kg/m^2^)	42.2 (41.1 – 43.6)	45 (41 – 47.5)	< 0.005
Diabetes Yes No	42 (48%) 45 (52%)	46 (49%) 47 (51%)	0.99

Continuous parameters were reported as median and interquartile ranges. Categorical variables were recorded as numbers and percentages

**Table 2:** Patients intraoperative characteristics

	Liraglutide *n* = *87*	No Liraglutide *n* = *93*	***p-value***
Adhesions Yes No	32 (37%) 55 (63%)	9 (10%) 84 (90%)	< 0.005
Operative Time (min)	95 (88–110)	82 (78–98)	< 0.05

Variables were recorded as numbers and percentages

**Table 3:** Post-operative complications according to Clavien-Dindo grade

	Liraglutide *n* = *87*	No Liraglutide *n* = *93*	***p-value***
***Clavien Dindo*** I II IIIa IIIb	10 (11%) 2 (2%) 3 (3.4%)	8 (8.6%) 1 (1%) 1 (1%)	0.13

Variables were recorded as numbers and percentages

## Discussion

Medical therapy with liraglutide, as an adjunct to a reduced-calorie diet and increased physical activity, can lead to a sustained and clinically relevant weight loss in overweight and obese patients. Liraglutide is generally well-tolerated, with gastrointestinal symptoms such as nausea, diarrhoea, vomiting, and abdominal fullness reported as the most frequent adverse events. However, a cohort study of Lando et al. [13] revealed an increased risk of pancreatitis with elevated serum amylase or/and lipase levels in patients treated with GLP-1 receptor agonist compared to nonusers. Other studies confirmed these data showing an increased risk of acute pancreatitis with an OR > 2 with liraglutide [14]. These data were supported by the study of Butler et al. [15] who found an increased release of pancreatic amylase with an hyperplastic or neoplastic effect on pancreatic duct glands, leading to obstruction of the small pancreatic ductless, resulting in acute pancreatitis, on animal models treated with liraglutide. Based on these data Hakim et al. demonstrated, for the first time, a significant incidence of intraoperative adhesions (22%) in patients with preoperative treatment with liraglutide compared to the control group [16]. The risk of acute pancreatitis linked to GLP-1 agonist was also reported by Pi-Sunyer et al. [17]. In this randomized control trial, gallbladder dysfunctions including higher incidence of cholelithiasis and cholecystitis, related to the decreased gall bladder motility in the liraglutide group, were more common after liraglutide compared to placebo (2.5% vs. 1.0%).

In our cohort of obese patients, intraoperative adhesions needed adhesiolysis, between the posterior gastric wall and the pancreas related to subclinical pancreatitis were reported in 37% of the patients treated with liraglutide before surgery compared to 10% of the control group, (*p* < 0.005) in line with data reported by Hakim et al. [16]. Although these findings were more evident in our study where only patients who reached the maximal dosage of Liraglutide were included, compared with the Hokim’s study, where also patients with incomplete or underdosed patients were included.

Furthermore, in our series, patients treated with liraglutide had a longer operative time compared to controls (*p* < 0.05). Different factors could be correlated with prolonged operative time, such as surgeon’s technical skill, resident involvement, case complexity [18, 19]. The prolonged operative time reported in patients treated with liraglutide in our study, could have been correlated with the higher incidence of intraoperative adhesion, since the operations were carried out by the same surgeon, however, despite the longer operative and complexity of the operations no significant differences in the postoperative complications were recorded.

In our analysis the preoperative use of GLP1 agonists showed improved weight loss at 12 months after surgery compared to the control group. Nevertheless, Herberg et al., in a published abstract at SOARD 2024 [20], reported in a retrospective analysis that patients who received pre-operative GLP1s had no significant difference in post-operative weight loss when compared with those that did not receive preoperative GLP1s. Although, this recent abstract didn’t precise the time of follow-up and the type of bariatric surgery; these data are crucial to understand the possible role of GLP1 agonist for weight loss after surgery.

The main limitations of this study are the single-centre experience, the limited follow-up, and its retrospective nature, which opens it to possible selection bias. Moreover, the operative surgeon knew if the patients had prior therapy with liraglutide or not. A future prospective, randomized, blinded, multicentre trial is indicated.

## Conclusion

Liraglutide has introduced a new option in the treatment of obese patients, favouring their weight loss and the relief of associated comorbidities and therefore its role as a bridge to surgery in obese patients has been supported by several studies. Nevertheless, as demonstrated in our study, this therapy could lead to subclinical pancreatitis favouring the onset of adhesion between the stomach and the pancreas which could make the gastric mobilization during LSG more technically demanding and risky.

## Data Availability

All data generated or analysed during this study are included in this article. Further enquiries can be directed to the corresponding author.
